# Air-Coupled Ultrasonic Detection of Surface Roughening and Ink Wettability

**DOI:** 10.3390/s26113334

**Published:** 2026-05-24

**Authors:** Guangya Li

**Affiliations:** 1 School of Microelectronics, Shanxi University of Electronic Science and Technology, Linfen 041000, China; jerry_9148@163.com; Tel.: +86-0351-15834162425; 2 State Key Laboratory of Extreme Environment Optoelectronic Dynamic Measurement Technology and Instrument, North University of China, Taiyuan 030051, China

**Keywords:** air-coupled ultrasound, C-scan imaging, non-destructive testing, paper aging, Biot theory, Rayleigh–Davies scattering, paper roughening, ink wettability

## Abstract

**Highlights:**

**What are the main findings?**

- The air-coupled ultrasonic (ACUT) system can quantitatively characterize the roughening degree of paper materials via the amplitude difference of ultrasonic transmission signals, and the results are highly consistent with the national standard GB/T 18739-2008.
- The ink wettability of paper can be evaluated by the pixel distribution of HSV color space in ACUT C-scan images, and the intermediate color pixel ratio is positively correlated with the ink roundness.

**What are the implications of the main findings?**

- The proposed non-contact, non-destructive testing method breaks through the limitations of traditional detection methods such as sample damage, strong subjectivity, and inability to large-area detection.
- This technology provides a reliable technical means for quality control and aging evaluation of paper cultural relics and high-grade paper, with broad application prospects in cultural heritage protection and paper industry.

**Abstract:**

In the field of traditional aging state evaluation of paper materials, traditional detection technologies such as ink drop method and chemical analysis have inherent limitations including sample damage, strong subjectivity, and inability to realize large-area detection. To address these problems, a non-contact and non-destructive testing method based on air-coupled ultrasonic technology was developed in this study, to achieve objective and quantitative characterization of paper roughening degree and ink wettability. The system adopted a LabVIEW-based host computer to control scanning and signal acquisition. Based on the propagation and scattering mechanism of ultrasound in the porous fiber structure of paper, the amplitude difference and pixel distribution of C-scan images were extracted as core characteristic parameters. The experimental results show that, with a 400 kHz air-coupled probe and 200 mm/s scanning speed, the roughening degree of paper can be quantitatively characterized by the amplitude difference of ultrasonic transmission signals. The amplitude difference increases significantly with the rise of water content, and the difference in roughening characteristics between newsprint and Xuan paper can be clearly distinguished. The ink wettability can be judged by the pixel distribution of the C-scan image: the higher the proportion of intermediate color pixels, the closer the ink circularity is to 1, and the better the ink wettability. All test results are highly consistent with the national standard GB/T 18739-2008. The constructed air-coupled ultrasonic testing system can provide reliable technical support for quality control and aging evaluation of paper cultural relics and high-grade paper by characterizing both surface roughening and internal porous structure (which are coupled during paper aging), without any contact or damage to the samples.

## 1. Introduction

Paper materials, as a fundamental carrier for human civilization evolution, production activities, and cultural communication, are widely used in key fields including printing and publishing, packaging and transportation, calligraphy and painting creation, long-term archival storage, and insulating media for high-voltage power equipment [1,2]. The quality of its microscopic fiber network and macroscopic mechanical/optical properties directly determines the service performance and ultimate storage life of paper. However, as a highly sensitive porous natural polymer composite system, paper is extremely vulnerable to environmental stresses such as drastic fluctuations of temperature and humidity, photo-oxidation, and microbial erosion during long-term storage and circulation [3].

Driven by these environmental stresses, the cellulose macromolecular network inside paper undergoes irreversible physical degradation and chemical decomposition. Chemically, the core aging pathways of paper mainly include acid hydrolysis and oxidative fracture of cellulose chains [4]. Hydrolysis reaction directly breaks the β-1,4-glycosidic bonds in cellulose, leading to a sharp decrease in the degree of polymerization (DPv) and the imbalance of microscopic topological ratio between crystalline and amorphous regions [4]. These underlying chemical changes are embodied in macroscopic physical properties as roughening damage and ink wettability degradation of paper. Roughening will cause irreversible internal stress release and warpage after fiber water absorption, resulting in paper brittleness and exponential decrease in mechanical tensile strength and folding endurance. Ink wettability degradation will change the hydrodynamic diffusion law of ink in the paper capillary network, which will seriously damage the edge sharpness of printing dots or the artistic halo effect of traditional calligraphy and painting. Therefore, developing high-throughput and non-destructive testing technology for key properties of paper, such as surface roughening degree and ink absorbency, has extremely high engineering value and scientific significance for ensuring industrial service quality, extending the preservation cycle of historical archives, and evaluating the health status of paper cultural relics.

At present, the mainstream evaluation methods for paper roughening and ink wettability degradation can be divided into two categories: traditional macroscopic evaluation based on physical morphology observation, and microscopic detection based on chemical composition analysis [5]. For physical morphology evaluation, the most representative methods are the ink drop method, gloss measurement method, and light transmittance detection method in accordance with GB/T 18739-2008 [6] Product of Geographical Indication-Xuan Paper [7]. The ink drop method is highly dependent on manual observation, and indirectly infers the uniformity of internal fiber pores by quantifying the perimeter, area and edge burr (circularity) of ink diffusion. Enomae et al. studied the surface roughening and gloss relaxation phenomenon of paper caused by moisture from the perspective of optical reflection, and pointed out that water molecules will break the hydrogen bond balance between fibers, leading to fiber expansion, stress relaxation, increased surface roughness and decreased gloss [8]. Takeru et al. proposed to characterize the evolution of internal microstructure and pore structure of paper by the attenuation characteristics of light transmittance [9]. However, traditional optical testing usually requires mechanical flattening or specific light source irradiation, which is easy to cause secondary irreversible physical indentation and photochemical damage to fragile aged fibers. Meanwhile, the ink drop method is a destructive contact test, which will cause permanent chemical pollution to samples, and its diffusion results are greatly affected by fluid surface tension and operating environment, with strong subjectivity and poor repeatability.

On the other hand, chemical analysis technology has unparalleled molecular-level resolution in the accurate quantification of paper aging mechanism. High-level paper aging evaluation widely relies on precision instruments such as size exclusion chromatography (SEC), Fourier transform infrared spectroscopy (FTIR), X-ray diffraction (XRD), and high performance liquid chromatography-mass spectrometry (HPLC-MS) [4]. For example, in the degradation study of historical documents with iron gall inks, researchers established an aging kinetic model based on environmental humidity by monitoring the auto-oxidation process of gallic acid via liquid chromatography [10]. In the aging monitoring of transformer insulating paper, the change of cellulose polymerization degree can be inverted with high precision by extracting the characteristic degradation product methanol dissolved in oil through gas chromatography [11]. Despite the high precision of chemical analysis, its engineering limitations are extremely prominent: almost all of these methods rely on destructive sampling, which is absolutely taboo for the protection of precious paper cultural relics and archives. Secondly, chemical analysis is essentially local point detection, which cannot achieve spatial continuous scanning of large-scale paper. Finally, the high equipment cost and complex sample pretreatment process make it impossible to apply in large-area real-time online detection of papermaking industrial lines.

In view of the limitations of traditional methods, Air-Coupled Ultrasonic Non-Destructive Testing (ACUT) has become an ideal cutting-edge technology in the field of material performance diagnosis, with its disruptive advantages of completely non-contact, non-destructive, and large-field macroscopic imaging [12]. ACUT completely abandons the liquid couplant required by traditional ultrasonic testing. By capturing the transmission, scattering and dispersion characteristics of sound waves propagating in complex media, it can highly sensitively reflect the defect distribution, pore connectivity and matrix uniformity inside the material. At present, this technology has made breakthroughs in the damage evaluation of aerospace composite materials, food texture identification, and integrity tomography of lithium-ion battery porous electrodes [13]. Gómez Álvarez-Arenas et al. first introduced broadband air-coupled ultrasonic spectroscopy into the porosity measurement of mineral paper, confirming that non-contact sound waves have excellent detection sensitivity for high-porosity paper-based materials [14]. However, the application of this technology in paper aging monitoring and cultural heritage protection still faces severe acoustic challenges. There is an extremely high acoustic impedance mismatch of several orders of magnitude between air and paper fibers, which will lead to nearly total reflection of ultrasonic energy at the interface, with a theoretical insertion loss as high as 120 dB to 160 dB [15]. In addition, the hydrogen bond fracture during paper aging will induce surface roughening and increased tortuosity of the internal porous network, which will further cause severe viscous dissipation and scattering attenuation of the already weak ultrasonic main beam.

To this end, this study built a dedicated high-precision scanning detection system based on air-coupled ultrasonic technology, aiming to break through the bottleneck of weak signal extraction under high impedance mismatch environment. We not only realized non-contact imaging of paper at the experimental level, but also systematically introduced Biot porous medium two-phase wave theory and Rayleigh–Davies random rough surface scattering model at the theoretical level, to deeply analyze the energy migration and dissipation law of ultrasound in heterogeneous paper materials. By establishing the mathematical correlation model between ultrasonic characteristic parameters (transmission amplitude difference, HSV pixel distribution of C-scan images) and paper roughening degree/ink wettability, this study aims to provide a novel theoretical framework and solid experimental support for non-destructive testing of paper key properties, and finally realize efficient, non-destructive and high-speed evaluation of paper cultural relics and industrial paper.

## 2. Materials and Methods

This section systematically introduces the theoretical basis of air-coupled ultrasonic testing for paper aging, the design of the dedicated testing system, sample preparation, and experimental procedures, to ensure the repeatability and reliability of the research.

### 2.1. Theoretical Basis of Air-Coupled Ultrasonic Testing for Paper Materials

#### 2.1.1. Propagation Characteristics of Ultrasound in Paper

The biggest physical challenge of air-coupled ultrasonic technology is the huge acoustic impedance mismatch between air and paper fiber medium, which directly determines the distribution of ultrasonic energy at the interface. Acoustic impedance Z, the core intrinsic parameter describing the acoustic properties of a medium, is defined as:

(1) Z=ρ·c

where ρ is the static density of the medium, and c is the sound velocity in the medium. When ultrasonic waves are incident perpendicularly to the air-fiber interface, let the incident sound pressure be $P_{0}$, reflected sound pressure be $P_{r}$, and transmitted sound pressure be $P_{t}$.

The sound pressure reflectivity r and transmittance t are defined as:

(2) $$r=P_{r}/P_{0}$$

(3) $$t=P_{t}/P_{0}$$

Based on the acoustic boundary conditions, the relationship between reflectivity/transmittance and the acoustic impedance of the two media $Z_{1}$ for air, $Z_{2}$ for paper fiber can be derived as:

(4) $$T_{12}=\frac{P_{t}}{P_{0}}=\frac{2\times Z_{2}}{Z_{2}+Z_{1}}$$

(5) $$R_{12}=\frac{P_{r}}{P_{0}}=\frac{Z_{2}-Z_{1}}{Z_{2}+Z_{1}}$$

Since the acoustic impedance of air is extremely low, while that of plant fiber is as high as 1.5 × 10^6^ Rayl ($Z_{2}$ ≫ $Z_{1}$), the transmittance and reflectivity can be approximated as:

(6) $$T_{12}=2,R_{12}\approx -1$$

Substituting the actual physical parameters, the single-interface sound pressure transmittance of 400 kHz ultrasound incident on Xuan paper is only 2.8 × 10^−2^, and the reflectivity is as high as 99.98%.

From the perspective of energy conservation, the sound intensity transmittance T is:

(7) $$T=\frac{4Z_{1}Z_{2}}{{\left(Z_{1}+Z_{2}\right)}^{2}}$$

The calculation shows that the energy loss of ultrasound passing through a single air-fiber interface exceeds 99.9%. In the transmission detection architecture, the sound wave needs to penetrate the “air-paper” front interface and “paper-air” rear interface, resulting in a theoretical insertion loss of more than 120 dB [15], which poses a strict challenge to hardware design and weak signal extraction.

In addition, the attenuation mechanism is also deeply related to the complex porous structure of paper. Xuan paper is not a dense homogeneous continuum, but a porous viscoelastic network composed of interwoven plant fiber skeleton and air micro-pores. Therefore, the simple uniform medium transmission model is invalid, and it is necessary to introduce the porous medium acoustic theory. This study adopts the classic Biot’s Theory of Poroelasticity [16], which describes the propagation of elastic waves in a fluid-saturated porous solid. According to this theory, ultrasonic waves will undergo mode conversion when entering paper, splitting into the Fast P1 Wave (carried by the solid skeleton, low attenuation) and the Slow P2 Wave (dominated by pore fluid displacement, high frequency dispersion and viscous attenuation) [17]. This theory provides a physical basis for understanding the energy dissipation of ultrasound in the paper pore network.

The essence of paper roughening is the breakage of inter-fiber hydrogen bonds caused by moisture, leading to fiber warpage and extreme disorder of internal pore morphology. To characterize the high-frequency viscous friction caused by slow wave propagation in distorted pores, this study further introduces the Johnson–Champoux–Allard (JCA) equivalent dynamic fluid model [17], which uses the complex form of equivalent dynamic density $\tilde{\rho }\left(\omega \right)$ to characterize the inertial effect and viscous dissipation in fluid-solid coupling:

(8) $$\tilde{\rho }(w)=\alpha_{\infty }\rho_{f}\left[1+\frac{\sigma \phi }{jw\rho_{f}\alpha_{\infty }}\sqrt{1+j\frac{4\alpha_{\infty }^{2}\eta \rho_{f}\omega }{\sigma^{2}\Lambda^{2}\phi^{2}}}\right]$$

where ω is the angular frequency of ultrasound,η is the dynamic viscosity of air, ϕ is the static flow resistance, $\rho_{f}$ is the static density of air, $\alpha_{\infty }$ is the geometric tortuosity factor, and Λ is the viscous characteristic length. When paper is aged and roughened, $\alpha_{\infty }$ increases significantly and Λ decreases sharply, leading to a substantial increase in the imaginary part of $\tilde{\rho }\left(\omega \right)$ (representing internal friction energy dissipation), and finally a sharp rise in the intrinsic absorption coefficient of ultrasound in paper.

In addition, fiber warpage leads to uneven paper surface and non-negligible surface scattering attenuation. Let the root mean square of paper surface roughness be $R_{q}$. According to the Davies’ model for rough surfaces [18], the amplitude of the coherent transmitted wave will show exponential attenuation with the increase in surface roughness:

(9) $$A=A_{0}e^{-2{\left(kR_{q}\cos\;\theta_{i}\right)}^{2}}$$

where k is the ultrasonic wave number, and $\theta_{i}$ is the incident angle for vertical transmission. With the increase in paper aging and roughening, the surface roughness increases, and a large amount of forward acoustic energy is converted into diffuse scattering, resulting in a sharp drop in the energy of the main sound beam captured by the receiving probe.

**Figure 1:**
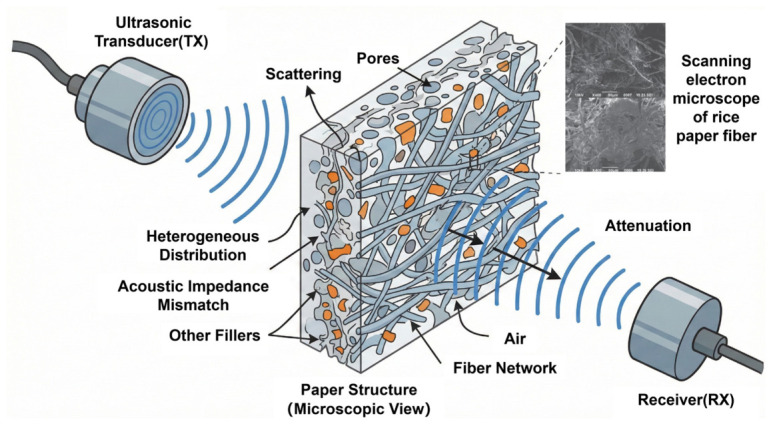
Ultrasonic transmission model for paper.

The macroscopic attenuation of transmission amplitude caused by the superposition of internal pore viscous absorption (Biot-JCA dissipation) and surface roughness diffuse scattering (Rayleigh–Davies dissipation) is the core physical basis for quantitative detection of paper aging and roughening in this study. The ultrasonic transmission paper model is shown in Figure 1.

#### 2.1.2. Detection Theory of Roughening Degree

**Figure 2:**
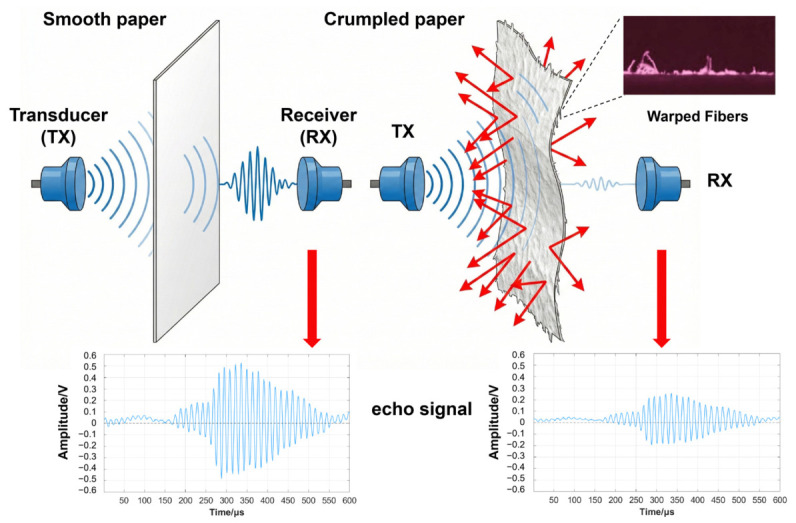
Echo characteristics of ultrasonic transmission paper.

Under dry conditions, the fibers of new paper are tightly fitted in a flat shape due to the pressing process. When encountering moisture, the fibers will absorb water and recover to a tubular structure, the inter-fiber hydrogen bonds will be broken, and the fibers will lift up from the paper surface, forming a multi-layer staggered interface structure of “air-fiber-air” [8]. This rough interface will disrupt the propagation path of ultrasound, leading to strong multiple reflections and diffuse scattering, and a significant reduction in transmission amplitude at the receiving end. The echo characteristics of ultrasonic transmission paper are shown in Figure 2.

This microscopic difference inside the paper can be transformed into the spatial distribution difference of ultrasonic transmission amplitude through the scanning system. In this study, the paper in natural dry state with intact structure was used as the reference control group, and the average amplitude difference of ultrasonic transmission signal in the whole scanning surface was defined as the reference benchmark of paper roughening degree:

(10) $$V_{s1}=V_{\max,1}-V_{\min,1}$$

where $V_{\max,1}$ and $V_{\min,1}$ are the average maximum and minimum ultrasonic amplitude of the dry reference group, respectively.

For the tested aged paper with different water content, the deviation difference between the average maximum amplitude of the tested group and the average minimum amplitude of the reference group was calculated, which represents the maximum acoustic attenuation amplitude caused by moisture:

(11) $$V_{s2}=V_{\max,2}-V_{\min,1}$$

where $V_{max,2}$ is the average maximum ultrasonic amplitude of the tested group under a specific water content.

By combining the intrinsic amplitude difference of the reference group and the deviation amplitude difference of the tested group, a dimensionless quantitative calculation formula of paper roughening degree $R_{d}$ was constructed:

(12) $$R_{d}=\frac{V_{S2}}{V_{S1}}\times 100\%$$

The value of $R_{d}$ is strictly positively correlated with the severity of fiber roughening. When $R_{d}\approx 100\%$ the paper structure is highly consistent with the dry reference group, and no obvious roughening occurs. When $R_{d}\gg 100\%$ the more significant the ultrasonic amplitude difference, the more serious the paper roughening degree.

#### 2.1.3. Detection Theory of Ink Wettability

For calligraphy, painting and high-precision printing, the ink wettability of paper is mainly determined by the uniformity of the internal fiber matrix, the softness of single fibers, and the three-dimensional distribution of micro-pores. When the fiber grid is evenly distributed and the capillary pore channels are reasonably arranged, the ink will be subjected to highly isotropic surface tension and capillary suction during diffusion, with smooth diffusion edges and excellent ink wettability [19]. On the contrary, disordered fibers, local hard blocks or voids will lead to extreme resistance mutation during ink diffusion, resulting in diffusion deformation, edge burrs and poor ink wettability.

When high-frequency ultrasound scans the paper sample vertically, the huge acoustic impedance mismatch between air and paper fibers will lead to continuous redistribution of ultrasonic energy between different physical phases. In the area with highly uniform fiber arrangement, the local acoustic impedance and porosity gradient are extremely small, and the ultrasonic transmittance remains basically constant, showing excellent spatial stability. In the area with disordered fibers, loose structure or voids, the ultrasound will encounter strong incoherent scattering, leading to significant and irregular fluctuation of forward transmittance. This difference in transmittance dispersion induced by fiber spatial heterogeneity can directly and non-contact reflect the ink wettability potential of paper.

During the scanning process, the system converts the spatial difference of transmittance into an intuitive pseudo-color image through C-scan two-dimensional imaging technology, and the color rendering is based on the HSV (Hue, Saturation, Value) color space mapping logic in LabVIEW. In the system setting, the intermediate color of the color gradient (cyan pixel band with a hue angle of about 120° in HSV space) is designated to represent the statistical median of the global ultrasonic amplitude. This median area means the most stable ultrasonic attenuation response and the optimal uniformity of the corresponding fiber structure.

In the subsequent processing, Matlab R2020a was used to perform color histogram analysis on the C-scan image, count the global proportion of each color pixel group, and quantify the concentration of intermediate color pixels in the whole scanning field of view. Meanwhile, to verify the accuracy of the acoustic evaluation system, the traditional optical circularity evaluation method specified in GB/T 18739-2008 was used for calibration. The calculation formula of circularity is:

(13) $$e=\frac{4\pi S}{A^{2}}$$

where S is the actual projected area of the single ink drop extracted after image binarization (measured by the number of pixels), and A is the peripheral perimeter of the ink area (total number of pixels of the closed contour edge). When the circularity coefficient is closer to 1, it indicates that the diffusion dynamics of ink in the paper pore network is more uniform, and the ink wettability of the paper is better.

### 2.2. Design of Air-Coupled Ultrasonic Testing System

**Figure 3:**
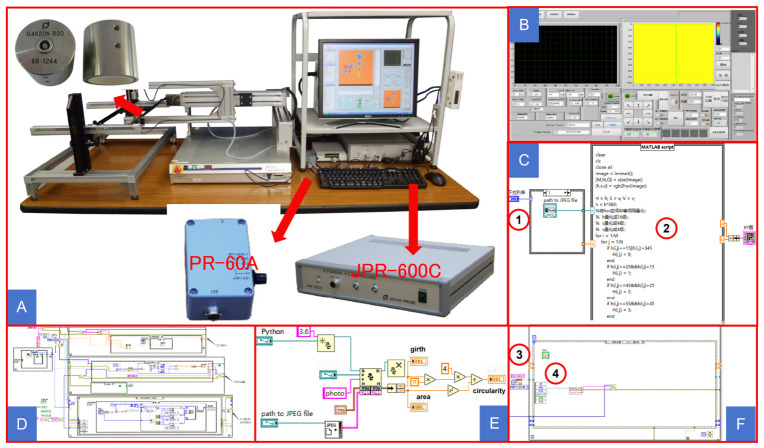
System software and hardware design: (**A**) actual hardware images of the system; (**B**) human–computer interface; (**C**) color histogram block diagram; ① 下拉列表: pull-down list, ② 将 hsv 空间非等量间隔化: Non-equidistant spacing of HSV space, h 量化成16级: Quantify into 16 levels, s 量化成4级: s Scaled into 4 levels, v 量化成4级: v Quantified into 4 levels; (**D**) ultrasonic C-scan module; (**E**) block diagram for calculating circularity; (**F**) block diagram of an arithmetic averaging filter. ③ 新建tcp主设备: Create a new TCP master device. ④ 源:source; 类型: type; 时间: time; 控件引用: Control Reference; 原值: original value; 新值: New Value.

To realize non-contact and non-destructive testing of paper-based materials, a dedicated air-coupled ultrasonic testing system was established in this study. As shown in Figure 3, the proposed system consists of a hardware system and a software system. The hardware system includes an ultrasonic transmitting and receiving module, a signal conditioning module, an air-coupled ultrasonic probe, a dual-axis motion scanning module, and a sample fixing platform. The software system is integrated in the host computer, which implements full-system parameter configuration, motion control, signal acquisition, data filtering, and visualization processing.

#### 2.2.1. Hardware System Design

The ultrasonic transmitting and receiving module is the core unit for ultrasonic excitation and signal acquisition, which adopts a JPR-600C ultrasonic generator and receiver. The device has a maximum sampling frequency of 100 MHz, is compatible with ultrasonic probes ranging from 30 kHz to 10 MHz, and can output stable ultrasonic excitation signals while receiving the transmitted ultrasonic signals that penetrate the sample.

The signal conditioning module is composed of a pre-amplifier and an electromagnetic compatibility (EMC) filter. A PR-60A pre-amplifier with an adjustable gain up to 60 dB is used to amplify the weak ultrasonic signal after penetrating the paper sample, so as to improve the signal-to-noise ratio of the received signal. An EMC filter is equipped in the ultrasonic C-scan system to suppress electromagnetic interference in the signal transmission link and further optimize the signal quality.

A 400 kHz air-coupled focused ultrasonic probe is selected as the acoustic excitation and sensing unit, which is adapted to the thin thickness characteristic of paper-based materials, and can realize high-resolution ultrasonic transmission detection of thin-layer paper samples.

The dual-axis motion scanning module is a two-dimensional motion system composed of a Delta DVP-ES2 programmable logic controller (PLC) and a 400 W servo motor. The system has an effective travel of 150 mm × 150 mm, an adjustable scanning speed ranging from 0 to 200 mm/s, and a positioning accuracy of 0.01 mm, which can ensure precise control of the scanning path of the ultrasonic probe during the detection process.

The sample placement platform is a flexible fixture that only fixes the 1 mm edge of the paper sample. This design avoids compression damage to the internal fiber structure of the paper during the fixing process, and ensures the flatness of the sample without displacement during the scanning process.

#### 2.2.2. Software System and Data Processing

The software system is developed based on LabVIEW, which is deployed on the host computer to realize human-computer interaction, full-process control of the detection, and data processing. The software system mainly includes a parameter configuration module, a synchronous motion and acquisition control module, a digital filtering module, and a data visualization and storage module.

First, the parameter configuration module provides a visual operation interface for users to set key detection parameters, including ultrasonic sampling frequency, signal gain, scanning range and step size. Meanwhile, the ultrasonic gating area is delimited in this module to screen effective transmitted ultrasonic signals. Second, the synchronous motion and acquisition control module realizes the synchronous triggering of the motion system and the ultrasonic generator and receiver. The module drives the servo motor to control the probe to move along the set scanning path, and synchronously triggers the ultrasonic transmitting and receiving module to complete signal excitation and acquisition.

Third, the digital filtering module adopts an arithmetic average filtering algorithm to perform noise reduction processing on the acquired ultrasonic signals, so as to eliminate random noise in the signals and improve the stability of the detection data.

Finally, the data visualization and storage module maps the processed ultrasonic signals and the probe position signals fed back by the encoder into HSV pseudo-color C-scan images in real time, and saves the original waveform data and C-scan images for subsequent qualitative and quantitative analysis of paper samples.

#### 2.2.3. Hardware and Software Signal Processing

**Figure 4:**
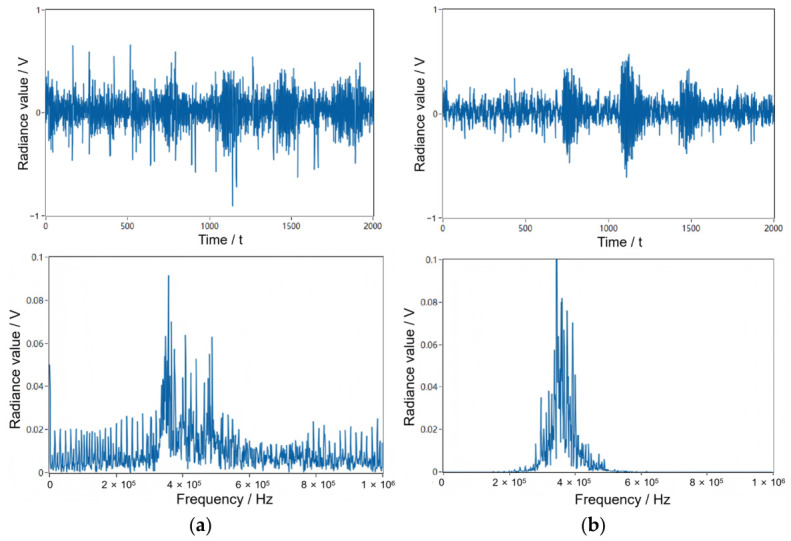
Hardware filtering: (**a**) time-domain and frequency-domain images of unfiltered signals; (**b**) time-domain and frequency-domain images after hardware filtering.

Hardware filtering utilizes EMC filtering. The application of this EMC filter significantly reduces abnormal noise during servo motor operation. Without using a preamplifier, the noise amplitude shows a marked decrease, thereby improving the signal-to-noise ratio. The filtering effect is shown in Figure 4:

The application of this EMC filter significantly reduces abnormal noise during servo motor operation. When no preamplifier is used, the noise amplitude decreases markedly, thereby improving the signal-to-noise ratio. However, due to substantial attenuation of air-coupled ultrasound signals in the air environment, preamplifiers are required to amplify ultrasound signals while simultaneously amplifying noise by several factors. This results in ultrasound signals being overwhelmed by noise, thereby diminishing the effectiveness of the EMC filter. Therefore, it is essential to implement software-level filtering techniques in addition to hardware filters to minimize the impact of electromagnetic noise generation.

The software filtering design employs the arithmetic mean filtering algorithm, which involves finding a value Y such that the sum of squared errors between this value and each sampled value X(k) (k = 1~N) is minimized. The calculation formula is:

(14) $$E=\sum_{K=1}^{N}{e^{2}}_{k}=\min\sum_{K=1}^{N}{\left(Y-X(K)\right)}^{2}$$

By the principle of limits, we have:

(15) $$Y=\frac{1}{N}\sum_{K=1}^{N}X(K)$$

**Figure 5:**
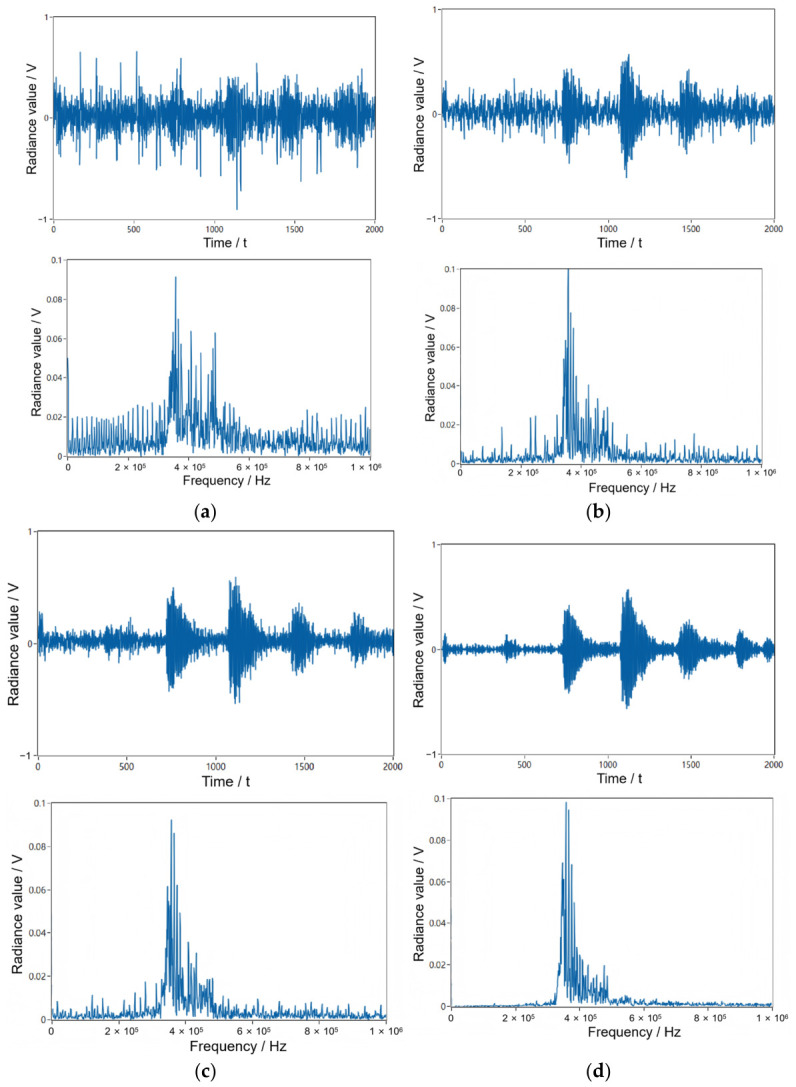
Time-domain and frequency-domain plots obtained by software filtering with different values of N: (**a**) initial waveform; (**b**) waveform at N = 4; (**c**) waveform at N = 10; (**d**) waveform at N = 16.

The image after signal processing by software is shown in Figure 5:

The observation in the figure above demonstrates that as the N value increases, signal spurs decrease and smoothness improves within the time domain range. In the frequency domain, the dominant frequency remains around 400 kHz while signal energy at other frequencies gradually diminishes. According to the table, when the motor is activated, the SNR reaches −5.4841 dB, making it impossible to distinguish effective signals from noise. After applying high-low pass filtering, the SNR shows slight improvement but still fails to meet detection requirements. The implementation of arithmetic averaging filtering yields a significant SNR enhancement, rising from 0.2325 dB with fourth-order averaging to 4.4571 dB with sixteenth-order averaging, representing a 9.9412 dB improvement. As shown in Table 1, this confirms that arithmetic averaging filtering effectively reduces electromagnetic noise interference on ultrasonic signals.

#### 2.2.4. Detection Workflow

The complete detection workflow of the established air-coupled ultrasonic testing system is as follows. First, environmental regulation and sample fixation. The laboratory environment was adjusted to room temperature with a relative humidity of 45% ± 5%, so as to avoid the interference of temperature and humidity fluctuations on ultrasonic propagation in air. The paper sample to be tested was fixed flat on the flexible fixture to ensure no displacement during the scanning process. Second, detection parameter configuration. Through the LabVIEW-based host computer software, key detection parameters including ultrasonic sampling frequency, signal gain, scanning range and scanning step were set, the ultrasonic gating area was delimited, and effective transmitted signals were screened.

**Figure 6:**
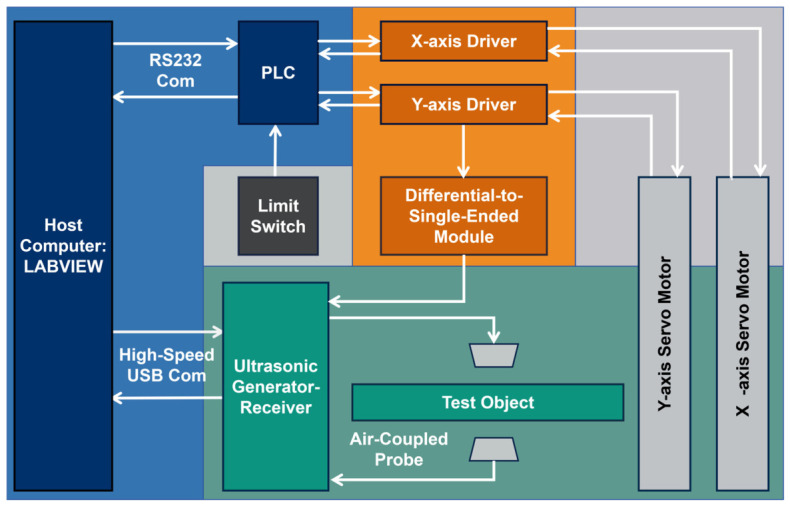
System detection flowchart.

The third phase involves scanning and signal acquisition. After parameter confirmation, the servo motor is activated to drive the ultrasonic probe along a Z-shaped path, synchronizing the ultrasonic transmitter and receiver to initiate ultrasonic signal emission. The ultrasonic signals penetrating the paper sample medium are captured by the receiving probe, amplified by a preamplifier, and fed back to the transmitter and receiver. Simultaneously, an arithmetic mean filtering algorithm is applied for software processing of the acquired signals. Data processing and storage are then completed. The processed ultrasonic signals and probe position signals transmitted via encoder are sent to the host computer through a high-speed USB interface. These signals are real-time mapped into HSV pseudocolor C-scan images, while original waveform data and C-scan images are stored for subsequent analysis. The system detection flowchart is illustrated in Figure 6.

#### 2.2.5. Data Processing and Feature Parameter Calculation

The raw data acquired by the system include the ultrasonic transmission signal (time-domain waveform) at each scanning grid point and its corresponding 2D coordinate. The following standardized calculation procedure was applied to extract the characteristic parameters of roughening degree and ink wettability.

For roughening degree evaluation:

For each A-scan signal (a single waveform) at a grid point, the maximum absolute amplitude within the predefined time gate was extracted using a peak-finding algorithm in LabVIEW. This value, denoted as $V_{max}(x,y)$, represents the local ultrasonic transmission intensity.

For a given paper sample under a specific moisture condition, the average maximum amplitude over the entire scanning area was calculated as $V_{max}=\frac{1}{N}\sum_{(x,y)\in S}V_{max}(x,y)$, where NN is the total number of scanning points. The average difference for the reference group (dry paper, moisture content of 0.22 g/m^3^) was further calculated as $\Delta {\bar{V}}_{ref}={\bar{V}}_{max,ref,dry}-{\bar{V}}_{min,ref,dry}.$ The roughening degree for a tested sample was then quantified using Equation (12) from Section 2.1.2: $R_{d}=\frac{{\bar{V}}_{\max,ref,dry}-{\bar{V}}_{\max,test,wet}}{\Delta {\bar{V}}_{ref}}\times 100\%$. For example, as shown in Table 2, for newsprint with a water content of 625 g/m^3^, ${\bar{V}}_{\max,test,wet}$ is 0.539 V, while ${\bar{V}}_{\max,ref,dry}$ is 0.913 V. With $\Delta {\bar{V}}_{ref}$ being 0.107 V, the calculated Rd is (0.913 − 0.539)/0.107) × 100% = 349.5%. A value significantly greater than 100% indicates pronounced fiber roughening.

The $V_{max}(x,y)$ values across the entire scan area were mapped to an HSV pseudo-color image. The hue channel, ranging from 0° to 360°, was linearly encoded to represent the normalized amplitude, with low amplitudes corresponding to high hue angles.

The C-scan image was imported into MATLAB. The hue channel (H) was extracted, and its histogram was computed with a bin size of 1°. The “intermediate color pixel ratio” was defined as the proportion of pixels whose hue angle falls within the cyan-green band (100° to 160°). This band was empirically determined to correspond to the median of the global ultrasonic amplitude distribution, indicating optimal fiber uniformity.

Ink Droplet Circularity: Following the GB/T 18739-2008 standard, the ink droplet image was binarized using Otsu’s method. The perimeter (A) and area (S) of the droplet were measured in pixels using the regionprops function in MATLAB. The circularity was then calculated as $C=4\pi S/A^{2}$. A value closer to 1 signifies a more uniform ink diffusion and better wettability.

## 3. Results

This section presents the experimental results of paper roughening degree characterization and ink wettability evaluation based on the air-coupled ultrasonic testing system, including the statistical analysis of ultrasonic signal parameters, quantitative calculation results of roughening degree, C-scan imaging results, and comparative verification results with the national standard method.

### 3.1. Characterization Results of Paper Roughening Degree

#### 3.1.1. Variation Law of Ultrasonic Amplitude with Water Content

**Figure 7:**
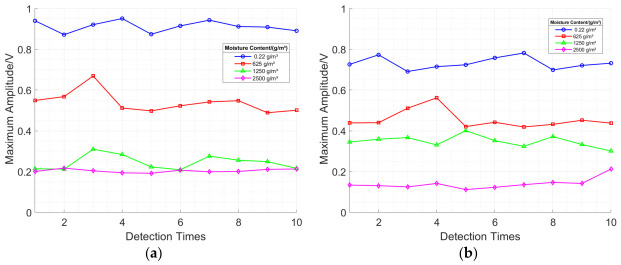
Variation of maximum ultrasonic amplitude of paper under different water contents: (**a**) newsprint; (**b**) Xuan paper.

The variation of the maximum ultrasonic amplitude of newsprint and Xuan paper under different water contents from 10 repeated scans is shown in Figure 7. The coefficient of variation for the maximum amplitude under each moisture content is less than 5%. It can be clearly seen that the maximum ultrasonic amplitude of both types of paper shows a continuous decreasing trend with the increase in water content, and the downward trend gradually slows down and enters a saturated state when the water content reaches a specific level. Among them, the amplitude of newsprint stabilizes in a low interval when the water content is close to 1250 g/m^3^, with little subsequent fluctuation. The saturation process of Xuan paper is slower, and the amplitude does not stabilize until the water content is close to 2500 g/m^3^.

To further reveal the stability and attenuation characteristics of ultrasonic signals during the roughening process, the average value and difference of the maximum waveform amplitude of paper with different materials and water contents were calculated from 10 scans, and the coefficient of variation and amplitude attenuation rate were supplemented. The specific parameters are shown in Table 2.

It can be seen from Table 2 that with the increase in water content, the attenuation characteristics of ultrasonic amplitude of the two types of paper are significantly different. The amplitude of newsprint drops sharply by 73.3% when the water content increases from 0.22 g/m^3^ to 1250 g/m^3^, and then the attenuation tends to be flat. While the attenuation of Xuan paper is closer to linear, with an attenuation rate of 80.9% at 2500 g/m^3^, which is higher than 77.8% of newsprint.

**Figure 8:**
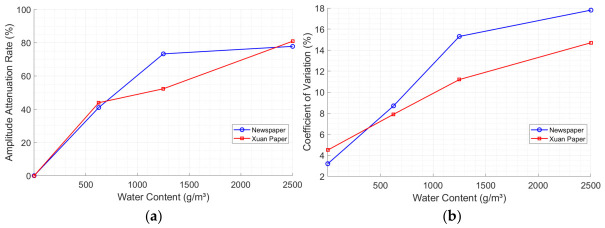
Characteristic parameter map of paper with different moisture content, (**a**) Amplitude Attenuation Rate, (**b**) Coefficient of Variation.

At the same time, the coefficient of variation of both types of paper increases continuously with the increase in water content. The coefficient of variation of newsprint rises from 3.2% to 17.8%, far exceeding the increase of Xuan paper from 4.5% to 14.7%. The reason is that the newsprint fiber is loose, and the inter-fiber bonding force is more easily destroyed after water absorption, resulting in a more prominent decrease in structural uniformity and a greatly enhanced randomness of ultrasonic scattering and reflection. The relatively dense fiber structure of Xuan paper makes the change of structural heterogeneity more gentle. The variation trends of amplitude attenuation rate and coefficient of variation with water content are shown in Figure 8.

#### 3.1.2. Quantitative Calculation Results of Roughening Degree

The roughening degree of paper was quantitatively characterized by the change rate of amplitude difference before and after wetting. The calculation results show that the roughening characteristics of newsprint and Xuan paper are significantly different.

When the water content of newsprint increases from 0.22 g/m^3^ to 1250 g/m^3^, the roughening degree jumps from 100% to 625.2%, and the amplitude difference increases by 5.9 times, indicating that its loose fiber structure expands and warps rapidly after water absorption. When the water content exceeds 1250 g/m^3^, the growth of roughening degree slows down, and it only rises to 663.6% at 2500 g/m^3^, because the fiber has reached water absorption saturation, and further water addition has no additional expansion effect.

**Figure 9:**
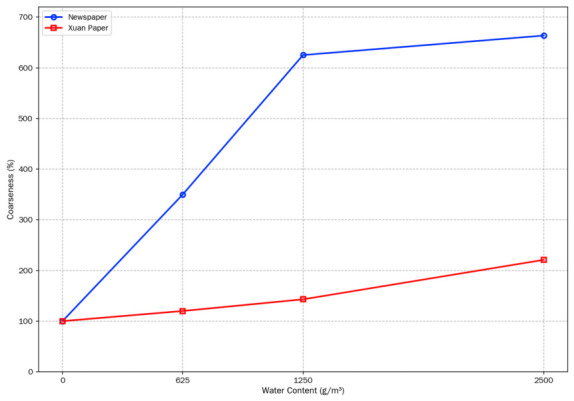
Variation in coarsening degree of different paper materials with moisture content.

The roughening degree of Xuan paper increases more gently with the growth of water content, which is 220.9% at 2500 g/m^3^, and the amplitude difference only increases by 1.2 times. The variation of paper roughening degree with water content is shown in Figure 9.

#### 3.1.3. C-Scan Imaging Results of Roughening Degree

**Figure 10:**
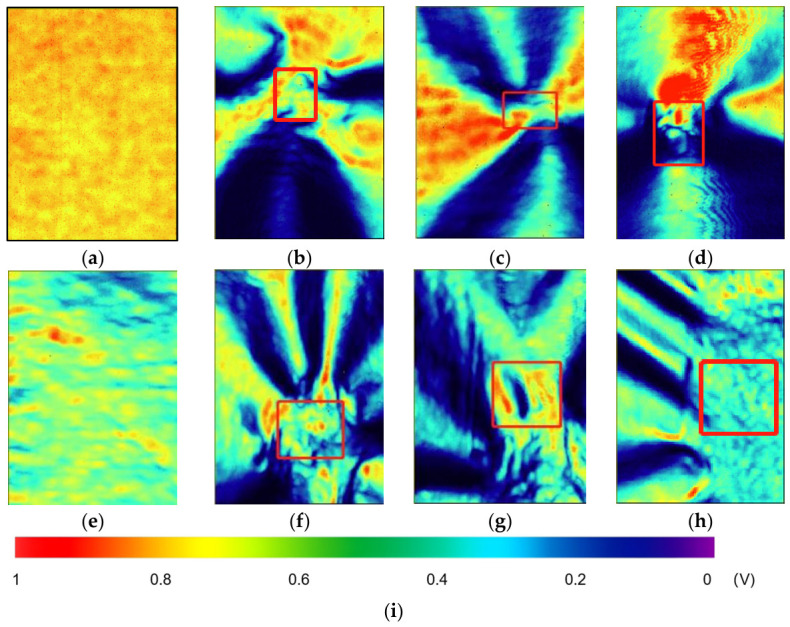
C-scan imaging results of roughening degree of different paper materials, The red box in the figure indicates the naturally dried area after water dripping, which is the region where the coarsening phenomenon occurs: (**a**) newsprint with 0.22 g/m^3^ water content; (**b**) newsprint with 625 g/m^3^ water content; (**c**) newsprint with 1250 g/m^3^ water content; (**d**) newsprint with 2500 g/m^3^ water content; (**e**) Xuan paper with 0.22 g/m^3^ water content; (**f**) Xuan paper with 625 g/m^3^ water content; (**g**) Xuan paper with 1250 g/m^3^ water content; (**h**) Xuan paper with 2500 g/m^3^ water content. (**i**) pseudo-color-Color bar corresponding to ultrasound amplitude values.

To intuitively present the spatial distribution characteristics of the roughened area, air-coupled ultrasonic C-scan imaging was performed on all samples. The imaging principle is to convert the ultrasonic amplitude into a pseudo-color signal, where red represents the high amplitude area and blue represents the low amplitude area. The C-scan imaging results of each group of samples are shown in Figure 10.

The C-scan image of the reference control group has uniform color without obvious red and blue spots, indicating regular fiber arrangement and no roughening. With the gradual increase in water content, the imaging color of the two types of paper begins to deviate from the uniform state, sporadic heterochromatic patches appear in the low water content stage, and in the medium and high water content stage, the heterochromatic area continues to expand, the distribution becomes more messy, and the color contrast and mottled sense are significantly enhanced. The C-scan imaging results are highly consistent with the calculation data of roughening degree, which can realize quantitative analysis and intuitive characterization of paper roughening degree.

### 3.2. Characterization Results of Paper Ink Wettability

#### 3.2.1. C-Scan Imaging and Pixel Analysis Results

**Figure 11:**
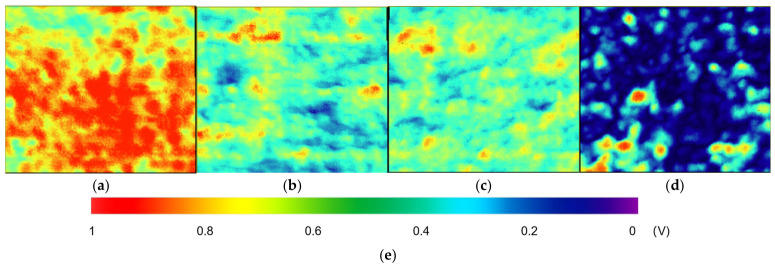
C-scan imaging results of ink wettability of Xuan paper with different thicknesses: (**a**) single layer; (**b**) double layer; (**c**) three layers; (**d**) four layers. (**e**) pseudo-color-color bar corresponding to ultrasound amplitude values.

The C-scan imaging results of laminated Xuan paper with different thicknesses are shown in Figure 11. It can be intuitively seen that the ultrasonic amplitude distribution of laminated Xuan paper with different thicknesses is significantly different. The imaging of the single-layer sample is dominated by red and yellow high-amplitude areas, while the four-layer sample presents scattered blue-black low-amplitude spots.

**Figure 12:**
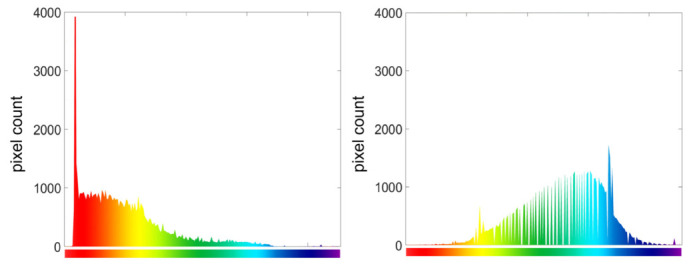

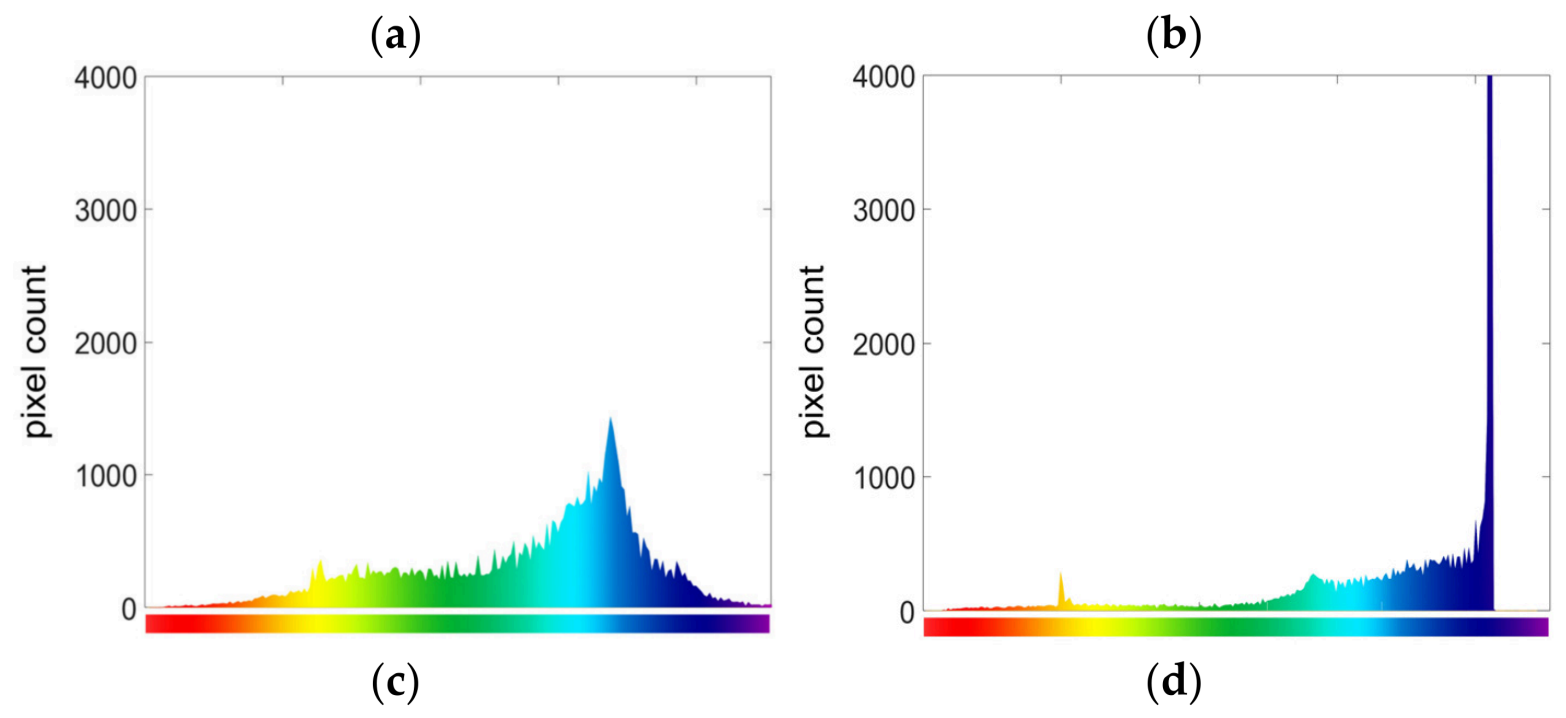
Color histogram of C-scan images of Xuan paper with different thicknesses: (**a**) single layer; (**b**) double layer; (**c**) three layers; (**d**) four layers.

To deeply analyze the influence of thickness on ultrasonic propagation and fiber structure, the average ultrasonic amplitude and amplitude standard deviation of laminated Xuan paper with different thicknesses were calculated. The C-scan images were imported into Matlab for HSV color space conversion, and the number of pixels corresponding to each color area was counted by setting the angle threshold of the Hue channel, to quantitatively obtain the fiber distribution characteristics of samples with different thicknesses. The color histogram of the C-scan images is shown in Figure 12. The statistical results of ultrasonic amplitude and histogram parameters are sorted out in Table 3.

From the analysis results of Figure 12 and Table 3, the average ultrasonic amplitude shows a linear downward trend with the increase of thickness, from 0.826 V of single-layer Xuan paper to 0.389 V of four-layer Xuan paper, with a decrease of 52.9%. This is because the increase in fiber layers leads to the extension of ultrasonic propagation path, the increase in reflection and scattering times, and then the aggravation of energy attenuation. The standard deviation of amplitude shows a trend of first decreasing and then increasing, reaching the minimum value in the three-layer Xuan paper, indicating that the fiber structure under this thickness is the most uniform, and the ultrasonic propagation stability is the best.

At the same time, with the increase in thickness, the peak angle of the histogram gradually increases from 35° to 175°, which is consistent with the color gradient of the HSV color space. The full width at half maximum first decreases to 45° and then increases to 73°, which further confirms that the pixel distribution of the three-layer Xuan paper is the most concentrated and the fiber structure uniformity is the best, while the pixel distribution of the single-layer and four-layer Xuan paper is relatively scattered, with more prominent structural heterogeneity.

#### 3.2.2. Ink Drop Verification Test Results

**Figure 13:**
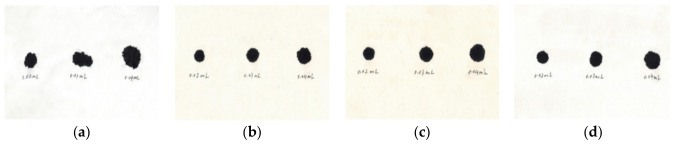
Actual images of ink dripping on Xuan paper with different thicknesses (**a**) single layer (**b**) double layer (**c**) triple layer (**d**) quadruple layer.

The actual ink drop images of laminated Xuan paper with different thicknesses are shown in Figure 13. It can be seen that the ink edge of the single-layer Xuan paper has serious burrs and uneven diffusion, the ink diffusion of the double-layer Xuan paper is relatively uniform but the edge has slight burrs, the ink of the three-layer Xuan paper is nearly circular without burrs, and the ink diffusion of the four-layer Xuan paper is uniform but the edge has slight bifurcation.

According to the traditional ink wettability detection method in GB/T 18739-2008, the perimeter and area parameters of the ink were extracted by ImageJ 1.53 software, and the circularity was calculated to quantify the ink wettability. The ink circularity of laminated Xuan paper with different thicknesses is shown in Table 4.

#### 3.2.3. Correlation Analysis Between Ultrasonic Characteristics and Ink Wettability

The pixel distribution characteristics of C-scan images and the results of ink wettability verification experiment were compared and analyzed, and the two showed a high internal consistency, as shown in Table 5.

Among them, the C-scan pixels of single-layer Xuan paper are concentrated in the low-angle area, reflecting its loose fiber structure and many local voids. This uneven structure leads to the disordered force of ink during diffusion, which is manifested as ink deformation and serious edge burrs in the ink wetting experiment, with a circularity of only 0.74 and poor ink wettability. The pixel distribution of double-layer Xuan paper is scattered in the medium and low angle region, corresponding to the medium degree of fiber interweaving. In the ink wetting experiment, the ink is elliptical with slight burrs on the edge, with a circularity of 0.84 and good ink wettability. The pixels of the three-layer Xuan paper are highly concentrated in the middle angle area, indicating uniform fiber distribution and optimal pore structure, so the ink is subjected to uniform force during diffusion, with a nearly perfect circle shape, no burrs, a circularity of 0.89, and excellent ink wettability. The pixels of the four-layer Xuan paper are biased to the medium and high angle area, and the fibers are locally stacked due to too many layers, which hinders the diffusion of ink. Finally, the ink is nearly circular, but the edge is slightly bifurcated, with a circularity of 0.77 and moderate ink wettability.

## 4. Discussion

This study established a dedicated non-contact and non-destructive testing system for paper aging based on air-coupled ultrasonic technology, and achieved quantitative characterization of paper roughening degree and ink wettability, which directly addresses the core technical pain points of sample damage, strong subjectivity, and poor repeatability in traditional paper performance detection methods. In this section, the intrinsic mechanism of the experimental results is interpreted in combination with classical acoustic theory, the reliability of the results is verified by comparing with existing studies, the core advantages and application value of the proposed method are discussed, and the limitations of the current research and corresponding future optimization directions are clarified.

From the perspective of physical mechanism, the significant attenuation of ultrasonic transmission signals in water-aged paper is the result of the joint action of two core acoustic dissipation mechanisms. The experimental phenomenon that ultrasonic amplitude decreases and coefficient of variation increases continuously with the rise of water content can be fully explained by the microstructure evolution of paper during water-induced aging. Water intrusion breaks the hydrogen bonds between cellulose fibers, causes fiber swelling and warpage, and eventually leads to the increase in surface roughness and the change of internal pore structure. On the one hand, according to the Rayleigh–Davies scattering model, the increased surface roughness will cause diffuse scattering of incident ultrasonic waves, resulting in a large loss of coherent main beam energy, which is the dominant factor of ultrasonic amplitude attenuation in the paper-air interface. On the other hand, the evolution of the internal pore structure of the paper increases the tortuosity of the pore channels, which enhances the viscous dissipation of air in the pores during ultrasonic propagation. This process is well supported by the Biot theory and JCA dynamic fluid model introduced in this study, which reveals the intrinsic correlation between pore structure evolution and ultrasonic energy loss in porous media. The superposition of interface scattering dissipation and pore viscous dissipation leads to the significant attenuation of ultrasonic transmission amplitude, which confirms that the ultrasonic amplitude difference can be used as a sensitive and reliable acoustic characteristic parameter to quantify the roughening degree of paper.

In terms of the difference in water-induced roughening characteristics between different paper materials, the experimental results show that newsprint is more prone to roughening deformation after water absorption than Xuan paper, which is determined by the inherent fiber structure characteristics of the two materials. Newsprint is made of short plant fibers with loose interwoven structure and weak inter-fiber bonding force. When encountering water, its hydrogen bond network is more easily destroyed, resulting in serious fiber warpage and structural heterogeneity. In contrast, Xuan paper is made of long plant fibers with a denser interwoven structure, which has better dimensional stability after water absorption, thus showing a lower roughening degree. This conclusion is highly consistent with the existing research on the water absorption and deformation characteristics of different paper materials, which further verifies that the air-coupled ultrasonic system built in this study can effectively distinguish the microstructure and performance differences of different paper materials [19,20].

For the evaluation of paper ink wettability, this study innovatively proposes a non-contact evaluation method based on the pixel distribution characteristics of HSV color space in ultrasonic C-scan images. The experimental results show that the proportion of intermediate color pixels in the C-scan image has a high consistency with the ink drop circularity measured by the national standard method GB/T 18739-2008, which confirms the reliability of this method. The intermediate color pixels correspond to the area with concentrated ultrasonic amplitude, which reflects the good uniformity of fiber and pore structure of the paper, and further determines the excellent ink wettability. Compared with the traditional ink drop method which is widely used in industrial production and cultural relic protection, this method has a revolutionary core advantage: it can realize the pre-evaluation of paper ink wettability without any contact with the sample, and will not cause any chemical pollution or physical damage to the paper. This unique advantage makes the method have irreplaceable application value in the protection of precious calligraphy, painting and paper cultural relics that cannot be sampled or contacted, as well as the on-line quality control of high-grade paper in industrial production lines.

Compared with the existing paper aging and performance detection methods, the air-coupled ultrasonic testing method proposed in this study has four significant core advantages, which break through the limitations of traditional technologies. First, it achieves completely non-contact and non-destructive detection. Unlike the ink drop method that causes chemical pollution to samples and the chemical analysis method that requires destructive sampling, this technology completely abandons coupling agents and contact operations, and will not cause any damage to paper samples, which is especially suitable for precious paper cultural relics and archives. Second, it realizes quantitative and objective characterization. Different from traditional optical detection and ink drop methods that are highly dependent on the subjective judgment of operators and have poor repeatability, this method extracts objective acoustic characteristic parameters, and the detection results are more reliable and repeatable. Third, it has the capability of large-area and high-resolution imaging. While the chemical analysis method can only realize point detection, the system built in this study can complete two-dimensional scanning of large-area paper with a scanning step of 0.1 mm, which can intuitively present the spatial distribution of local roughening and structural defects through C-scan images. Fourth, it has high detection efficiency. Compared with the traditional chemical analysis method that requires complex sample pretreatment and long test cycle, the maximum scanning speed of this system can reach 200 mm/s, which can complete the detection of large-size samples in a short time, and has the potential of on-line detection in industrial production lines.

Meanwhile, the current research still has three main limitations that need to be further optimized in follow-up studies. First, the detection performance for ultra-fragile aged paper needs to be improved. For extremely fragile paper cultural relics with severe aging and structural damage, the existing system still needs to further optimize the signal-to-noise ratio of weak signal extraction, including the improvement of probe parameters and signal processing algorithms, to adapt to the detection of such samples. Second, the comprehensive evaluation of paper aging needs to be expanded. This study mainly focuses on the two indicators of roughening degree and ink wettability. In the follow-up research, more acoustic characteristic parameters such as ultrasonic velocity, spectrum characteristics and nonlinear parameters will be extracted to realize the comprehensive evaluation of multiple aging indicators including paper mechanical strength, polymerization degree and fiber degradation degree. Third, the quantitative correlation between ultrasonic parameters and paper aging degree needs to be established. In this study, gradient water content is used to simulate the paper roughening process. In the follow-up, accelerated aging experiments under different environmental conditions will be carried out to establish an aging kinetic model between ultrasonic characteristic parameters and paper aging time and aging degree, so as to realize the residual life prediction of paper materials.

We recognize that direct visualization of the fiber microstructure (e.g., via SEM or optical microscopy) would strengthen the correlation between ultrasonic parameters and physical structural changes. In this study, we prioritized non-destructive testing and preserved the samples intact. To compensate, we have provided detailed references [8,19,20] that report microscopic images of similar paper materials under different moisture conditions, showing fiber swelling, warpage, and pore structure evolution. These reported microstructures are fully consistent with our ultrasonic findings. In future work, we will conduct parallel destructive and non-destructive tests on separate but identical samples to establish a direct microstructure–acoustic parameter library.

Overall, the air-coupled ultrasonic non-destructive testing method proposed in this study reveals the intrinsic correlation between paper microstructure evolution and ultrasonic propagation characteristics, breaks through the technical bottlenecks of traditional paper detection methods, and provides a new technical approach for the aging evaluation and health monitoring of paper materials.

## 5. Conclusions

In this study, a dedicated non-contact and non-destructive testing system for paper aging based on air-coupled ultrasonic technology was established, which successfully realized the quantitative characterization of paper roughening degree and ink wettability, and effectively overcame the core limitations of sample damage and strong subjectivity in traditional paper performance detection methods.

The key experimental findings of this study are summarized as follows. First, in the roughening degree detection experiment, as the water content of newsprint and Xuan paper increased from 0.22 g/m^3^ to 2500 g/m^3^, the ultrasonic amplitude difference increased from 0.107 V to 0.71 V and from 0.268 V to 0.592 V, respectively. The maximum roughening degree of newsprint reached 663.6%, which was much higher than 220.9% of Xuan paper, revealing that newsprint fibers are more prone to water-induced swelling and roughening deformation. The spatial heterogeneity of the roughened area was also intuitively presented via the gradually disordered and mottled color distribution in the ultrasonic C-scan images. Second, in the ink wettability evaluation experiment, the innovative method based on the pixel distribution characteristics of HSV color space in C-scan images was verified to be reliable for characterizing the uniformity of paper fiber and pore structure. The three-layer Xuan paper had the highest concentration of intermediate color pixels, with a corresponding ink drop circularity of 0.89 and the optimal ink wettability; while the single-layer and four-layer Xuan paper showed poor wettability with circularity of only 0.74 and 0.77, respectively. This result was highly consistent with the evaluation results in the national standard GB/T 18739-2008, which further confirmed the accuracy and reliability of the proposed method.

Compared with traditional paper detection methods and existing related studies, the air-coupled ultrasonic testing method proposed in this study has significant core advantages, including completely non-contact and non-destructive operation without sample contamination, quantitative and objective characterization with high repeatability, large-area high-resolution two-dimensional imaging with 0.1 mm scanning accuracy, and high detection efficiency with a scanning speed up to 200 mm/s. It breaks through the technical bottlenecks of traditional methods, provides a new technical approach for the aging evaluation and health monitoring of paper materials, and has important application value in the fields of precious paper cultural relics protection, archival preservation, and industrial quality control of high-grade paper.

Meanwhile, the current research still has some limitations to be addressed in follow-up work. First, the signal-to-noise ratio of the system for weak signal extraction will be further optimized, and the probe parameters and signal processing algorithm will be improved to adapt to the detection of ultra-fragile aged paper relics with severe structural damage. Second, more acoustic characteristic parameters including ultrasonic velocity, spectrum characteristics and nonlinear parameters will be extracted to realize the comprehensive evaluation of multiple aging indicators of paper, such as mechanical strength, polymerization degree and fiber degradation degree. Third, accelerated aging experiments under different environmental conditions will be carried out to establish an aging kinetic model between ultrasonic characteristic parameters and paper aging time/degree, so as to realize the residual life prediction of paper materials.

Overall, the air-coupled ultrasonic non-destructive testing method proposed in this study provides scientific and practical technical support for the quality control, storage evaluation, and performance optimization of paper materials, and has broad application prospects in related fields.

## Figures and Tables

**Table 1:** Three filter noise reduction signal-to-noise ratios.

	Initial	High-Low Pass Filtering	N = 4	N = 10	N = 16
SNR/dB	−5.4841	−3.2729	0.2325	2.0634	4.4571

**Table 2:** Statistical table of ultrasonic amplitude parameters of newsprint and xuan paper under different water contents.

Paper Type	News Paper				Xuan Paper			
Moisture Content/(g/m^3^)	0.22	625	1250	2500	0.22	625	1250	2500
Average Maximum Amplitude/V	0.913	0.539	0.244	0.203	0.732	0.411	0.349	0.140
Average Difference/V	0.107	0.374	0.669	0.71	0.268	0.321	0.383	0.592
Coefficient of Variation/%	3.2	8.7	15.3	17.8	4.5	7.9	11.2	14.7
Amplitude Attenuation Rate/%	0	41.0	73.3	77.8	0	43.9	52.3	80.9

**Table 3:** Ultrasonic amplitude and histogram parameters of Xuan paper with different thicknesses.

Test Sample	Thickness/mm	Average Amplitude/V	Amplitude Standard Deviation/V	Peak Angle of Histogram/°	FWHM of Histogram/°
Single-layer laminated Xuan paper	0.1	0.826	0.157	35	82
Double-layer laminated Xuan paper	0.2	0.643	0.102	85	67
Triple-layer laminated Xuan paper	0.3	0.517	0.068	130	45
Quadruple-layer laminated Xuan paper	0.4	0.389	0.115	175	73

**Table 4:** Roundness of ink droplets in different thicknesses of ink-wicking layers.

Test Sample	Perimeter/Pixels	Area/Pixels	Circularity
Single-layer laminated Xuan paper	1682	166,584	0.74
Double-layer laminated Xuan paper	1402	151,457	0.84
Triple-layer laminated Xuan paper	1518	163,284	0.89
Quadruple-layer laminated Xuan paper	1620	160,890	0.77

**Table 5:** Correlation between C-scan pixel distribution and ink wettability of laminated Xuan paper.

Test Sample	Thickness/mm	Pixel Concentration Range	Main Pixel Proportion/%	Circularity	Ink Moisturizing Property
Single-layer laminated Xuan paper	0.1	Low-angle (0–50°)	Red-yellow: 60	0.74	Poor
Double-layer laminated Xuan paper	0.2	Medium-low angle (50–100°)	Yellow-cyan: 52	0.84	Good
Triple-layer laminated Xuan paper	0.3	Medium-angle (100–160°)	Cyan-green: 75	0.89	Excellent
Quadruple-layer laminated Xuan paper	0.4	Medium-high angle (160–200°)	Blue-green: 48	0.77	Moderate

## Data Availability

The original contributions presented in this study are included in the article. Further inquiries can be directed to the corresponding author.
